# Association between watching eating broadcasts like *mukbang* and *cookbang* and generalized anxiety disorder among Korean adolescents

**DOI:** 10.1186/s12888-024-05957-z

**Published:** 2024-07-30

**Authors:** Jung-Hwan Kim, Jinhyun Kim, Suk-Yong Jang, Eun-Cheol Park

**Affiliations:** 1 Department of Health Informatics and Biostatistics, Graduate School of Public Health, Yonsei University, Seoul, Republic of Korea; 2 Department of Preventive Medicine, Yonsei University College of Medicine, Seoul, Republic of Korea; 3 Department of Family Medicine, Yonsei University College of Medicine, Seoul, Republic of Korea; 4 Department of Psychiatry, Yonsei University College of Medicine, Seoul, Republic of Korea; 5 Institute of Health Services Research, Yonsei University, Seoul, Republic of Korea; 6 Department of Healthcare Management, Graduate School of Public Health, Yonsei University, Seoul, Republic of Korea

**Keywords:** Generalized anxiety disorder, GAD-7, Mukbang and cookbang, KYRBS, Adolescents

## Abstract

**Background:**

Anxiety disorders are common during adolescence; therefore, detecting anxiety disorders among adolescents and providing appropriate treatment are crucial. Studies have suggested that watching online audiovisual broadcasts like *mukbang* and *cookbang* (hereafter *mukbang*), where hosts eat or cook food, may influence anxiety disorders. However, there is insufficient research on the association between watching *mukbang* and generalized anxiety disorder (GAD). Therefore, we investigated the association between watching *mukbang* and GAD among Korean adolescents.

**Methods:**

We analyzed 51,764 adolescents who participated in the 2020 Korea Youth Risk Behavior Web-Based Survey (KYRBS). The participants were asked how frequently they watched *mukbang* per week over the past 12 months. Anxiety disorders were assessed using the generalized anxiety disorder-7 (GAD-7) questionnaire. A multiple logistic regression analysis was performed after adjusting for confounding variables.

**Results:**

The prevalence of GAD was higher among adolescents who watched *mukbang* compared to those who did not (aOR: 1.100, 95% CI: 1.026–1.180, *P* = 0.008 in male participants; aOR: 1.090, 95% CI: 1.003–1.185, *P* = 0.042 in female participants). The frequency of watching *mukbang* showed a dose-dependent relationship with a greater likelihood of GAD in female adolescents.

**Conclusion:**

This study’s results showed that watching *mukbang* is associated with GAD in Korean adolescents. Proper interventions for mental health are needed for adolescents who watch mukbang.

**Supplementary Information:**

The online version contains supplementary material available at 10.1186/s12888-024-05957-z.

## Introduction

Anxiety disorders are common during adolescence [1]. Anxiety is a negative emotion that arises from fear, threats, or stress [2]; while short-term anxiety can be beneficial and help people solve problems and prepare for danger [3], long-term anxiety can develop into an anxiety disorder. Anxiety disorders in adolescence are chronic conditions that can be accompanied by other psychiatric illnesses such as depression [4]; therefore, it is critical to prevent anxiety disorders in adolescents. A lifetime prevalence of anxiety disorders is estimated at 26.1% in male students and 38.0% in female students. Among adolescents with anxiety disorders, 8.3% develop severe impairment [5]. As anxiety disorders can have a negative impact on adolescents’ mental health, the early management of anxiety disorders is important to reduce the burden of the disease [6].

*Mukbang* (eating broadcast)—a portmanteau of *meokneun* (eating) and *bangsong* (broadcast) —is a type of online eating show, while *cookbang* is an online cooking show. *Mukbang* and *cookbang* (hereinafter referred to as *mukbang*) originated in South Korea [7] in the late 2000s and has become widely popular along with the increasing popularity of single broadcasting media. An estimated 38% of Koreans watch *mukbang* online [8]. Over the past decade, *mukbang* has been disseminated worldwide and gained popularity through social media platforms such as YouTube [9]. With the increasing popularity of *mukbang*, interest in the impact of *mukbang* consumption is also growing.

Studies have shown that watching *mukbang* can be addictive, and there are concerns that it could lead to poor mental health outcomes [10]. The dissemination of *mukbang* through media channels is known to encourage overeating, and young people in particular are affected by food-related broadcasts [11, 12]. To increase viewership, *mukbang* creators not only overeat within a set time but also eat unhealthy foods or try risky foods [13]. Adolescent viewers may consume addictive foods through the imitation effect, develop poor eating habits, and sometimes develop eating disorders [14].

Despite the suggestion that *mukbang* may influence anxiety disorders [15], there is insufficient research on the association between watching *mukbang* and generalized anxiety disorder (GAD); furthermore, no research has been conducted on GAD in adolescents. Understanding the impact of *mukbang* on GAD could help to improve the mental health status of adolescents. This study investigates the association between watching *mukbang* and GAD in Korean adolescents.

## Methods

### Study population and data

Data were collected from the 2022 Korea Youth Risk Behavior Web-Based Survey (KYRBS), which has been conducted by the Korea Disease Control and Prevention Agency (KDCA) since 2005. The KYRBS is an anonymous, self-report online survey conducted nationwide among middle and high school students. This study provides fundamental data to evaluate the health of Korean adolescents and formulate health-related policies [16].

**Fig. 1:**
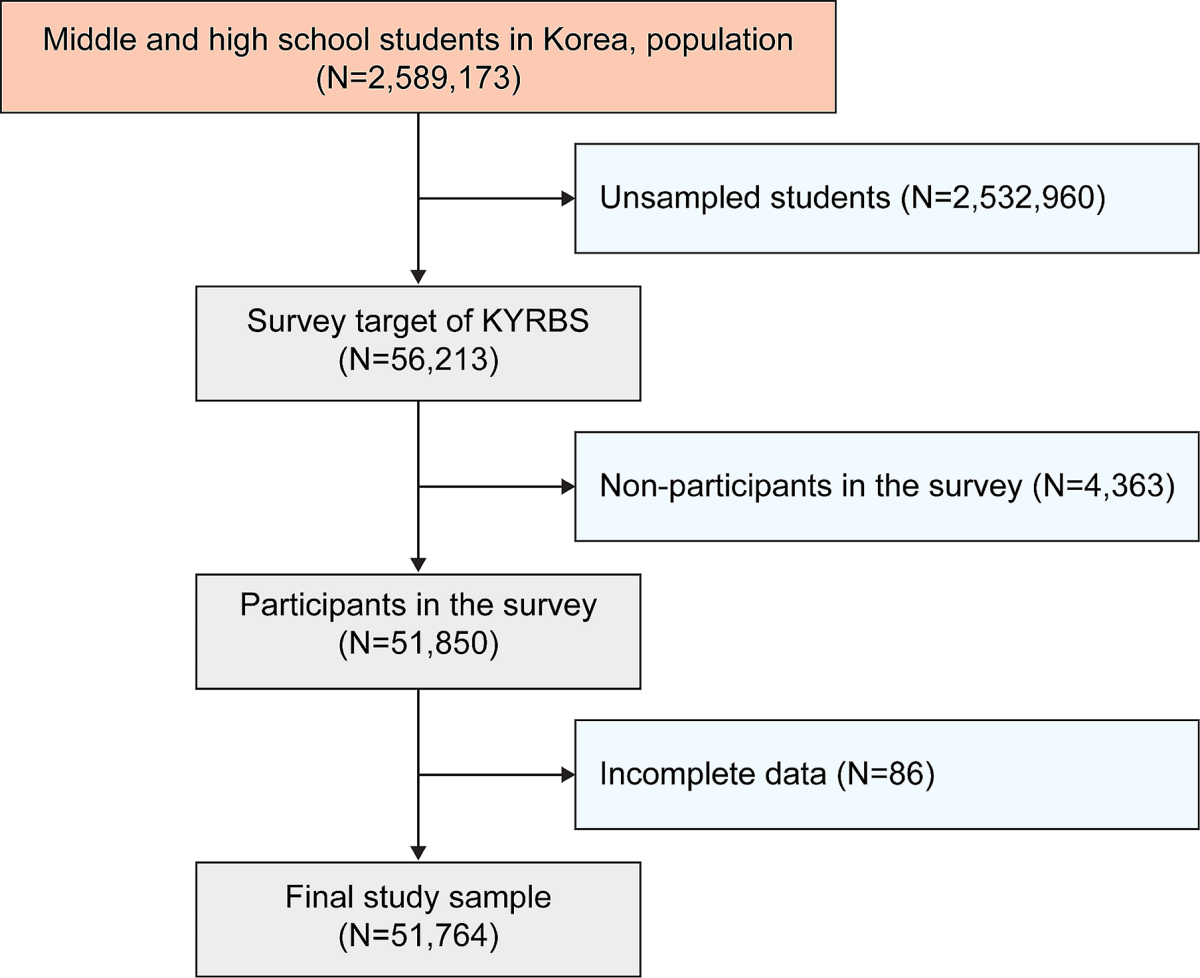
Flowchart of the study population

This study adhered to the principles of the Declaration of Helsinki, and the participant recruitment procedure was approved by Statistics Korea (Approval no.: 117,058) as a national official statistic. Research on the topic of *mukbang* began in 2022. To maintain the representativeness of Korean adolescents, this study selected a total of 400 middle schools and 400 high schools by sampling from all regions and types of schools in Korea under the supervision of the KDCA. The sample schools were selected using permanent random sampling within each stratum [17]. This survey investigated socioeconomic status and health-related behaviors through health-related interviews or assessments using 114 questions [18]. The study population consisted of 2,589,173 Korean middle and high school students. The response rate of the survey was 92.2%, resulting in the exclusion of 4,363 non-participants. Non-participation occurred due to teachers’ heavy workload related to the survey and their inability to use computer labs. Additionally, 86 participants were excluded owing to missing data. The final sample included 51,764 students (26,354 male and 25,410 female) (Fig. 1).

### Measures

#### Generalized anxiety disorder-7

The generalized anxiety disorder-7 (GAD-7) questionnaire is an online questionnaire comprising seven items. It is used to screen for anxiety disorders and is included in the Diagnostic and Statistical Manual of Mental Disorders (DSM- IV, 4th edition) [19]. The GAD-7 has been confirmed as a reliable tool for detecting GAD [20]. Each item is rated between 0 and 3, with a maximum combined score of 21 [21]. Studies have verified the optimal sensitivity and validity of the GAD-7 [22]. Cronbach’s alpha for the Korean version of GAD-7 was 0.915 with a cutoff score of 5. Positive predictive value, negative predictive, sensitivity, and specificity of GAD-7 were 46.3%, 92.4%, 78.1%, and 74.6%, respectively. So, if a GAD-7 score was ≥ 5, participants were assumed to have an anxiety disorder [23] In this study, the Cronbach’s α indicating the internal consistency of the scale was 0.91.

#### Mukbang and cookbang

Adolescents were surveyed to assess their frequency of viewing *mukbang* over the past 12 months. Answer options were: 1 (“Not at all”), 2 (“Less than once/month”), 3 (“Once–three times/month”), 4 (“Once–twice/week”), 5 (“Three–four times/week”), 6 (“Five–six times/week”), and 7 (“Daily”). Participants were categorized into two groups: “Never watching” (those who chose option 1) and “Ever watching” (those who chose options 2–7). To examine the dose response, the “Ever watching” group was further subdivided into four groups based on their frequency of viewing *Mukbang*: “Never” (option 1), “Rarely” (options 2–3), “Often” (options 4–6), and “Always” (option 7).

#### Covariates

Sociodemographic, health-related, and psychosocial factors were assessed as covariates. Sociodemographic factors included gender, age, type of family, socioeconomic status, and grade; health-related factors included physical activity, drinking, smoking, average sleep duration, and body mass index (BMI); and psychosocial factors included perceived stress level, self-reported health condition, and suicidality.

Age was divided into two groups based on the school the students attended: middle school (12–15 years old) and high school (16–18 years old). Family structure was classified as one parent, both parents, or none. Socioeconomic status, perceived stress levels, academic achievement, and self-reported health status were categorized into three groups.

Sleep duration was categorized into three groups: less than 6 hours’ sleep, 6–8 hours’ sleep, and more than 8 hours’ sleep [24]. Sufficient physical activity was defined as occurring more than 5 days a week, otherwise it was classified as insufficient based on the participants’ response to the item: “How many days a week did you engage in physical activity over 60 minutes per day?” We calculated the participants’ BMI and percentile values for height and weight. Participants were classified as underweight if their BMI percentile was < 5th percentile, normal weight if it was within the ≥ 5th and < 85th percentile, overweight if it was within the ≥ 85th and < 95th percentile, and obese if it was ≥ 95th percentile [25]. Suicidalities included suicidal ideation, suicide plans, and suicide attempts over the past year.

### Statistical analysis

The chi-square test was used to examine the statistical differences within each categorized group. All the analyses were stratified by sex. We conducted a multiple logistic regression after adjusting for confounding variables to investigate the association between *mukbang* watching and GAD-7. Subgroup analyses were conducted to examine the association between the frequency of watching *mukbang* using the GAD-7. The results were expressed as adjusted odds ratios (aOR) with 95% confidence intervals (CIs). The analyses were conducted using the stratified sampling variables (strata) and weighted variables recommended by the KYRBS. SAS version 9.4 was used for the data analysis, and the statistical significance level in all the analyses was set to *P* < 0.05.

## Results

Table 1 shows the baseline characteristics of the study population stratified by sex. Among the total 51,764 participants, 26,354 (50.9%) were male and 25,410 (49.1%) were female; 16,882 (64%) males and 20,142 (79.3%) females watched *mukbang.* Among all the adolescents who watched *mukbang*, GAD was present in 5,145 (30.5%) male and 8,598 (42.7%) female participants.

**Table 1:** Baseline characteristics of the study population according to GAD-7

Variables		Male (*N* = 26,354)					Female (*N* = 25,410)				
		GAD-7 < 5		GAD-7 ≥ 5		*p*-value	GAD-7 < 5		GAD ≥ 5		*p*-value
		*N*	(%)	*N*	(%)		*N*	(%)	*N*	(%)	
**Mukbang and Cookbang**						< 0.001					< 0.001
	Never watching	6,824	(72.0)	2,648	(28.0)		3,206	(60.9)	2,062	(39.1)	
	Ever watching	11,737	(69.5)	5,145	(30.5)		11,544	(57.3)	8,598	(42.7)	
**Age**						< 0.001					0.649
	12–15	11,002	(71.9)	4,302	(28.1)		8,679	(58.2)	6,242	(41.8)	
	16–18	7,559	(68.4)	3,491	(31.6)		6,071	(57.9)	4,418	(42.1)	
**Family structure**						0.314					< 0.001
	Both parents	12,926	(70.4)	5,447	(29.6)		11,785	(58.0)	8,530	(42.0)	
	Single parent	747	(68.7)	340	(31.3)		578	(52.1)	531	(47.9)	
	No parent	4,888	(70.9)	2,006	(29.1)		2,387	(59.9)	1,599	(40.1)	
**Alcohol status**						< 0.001					< 0.001
	Non-drinker	11,755	(72.7)	4,417	(27.3)		11,054	(61.3)	6,973	(38.7)	
	Past drinker	6,554	(67.3)	3,178	(32.7)		3,591	(50.2)	3,558	(49.8)	
	Current drinker	252	(56.0)	198	(44.0)		105	(44.9)	129	(55.1)	
**Smoking status**						< 0.001					< 0.001
	Non-smoker	16,618	(71.4)	6,662	(28.6)		14,157	(59.1)	9,803	(40.9)	
	Current smoker	1,943	(63.2)	1,131	(36.8)		593	(40.9)	857	(59.1)	
**Academic achievement**						< 0.001					< 0.001
	High	7,416	(71.2)	2,997	(28.8)		5,752	(59.9)	3,855	(40.1)	
	Middle	5,523	(72.7)	2,079	(27.3)		4,717	(60.0)	3,144	(40.0)	
	Low	5,622	(67.4)	2,717	(32.6)		4,281	(53.9)	3,661	(46.1)	
**Sleep duration**						< 0.001					< 0.001
	More than 8 h	2,348	(78.5)	642	(21.5)		1,102	(70.1)	470	(29.9)	
	6–8 h	7,621	(74.1)	2,659	(25.9)		5,208	(63.6)	2,980	(36.4)	
	Less than 6 h	8,592	(65.7)	4,492	(34.3)		8,440	(53.9)	7,210	(46.1)	
**Perceived stress level**						< 0.001					< 0.001
	High	4,289	(45.6)	5,118	(54.4)		4,101	(34.3)	7,845	(65.7)	
	Middle	9,174	(79.9)	2,301	(20.1)		7,587	(74.8)	2,553	(25.2)	
	Low	5,098	(93.2)	374	(6.8)		3,062	(92.1)	262	(7.9)	
**Physical activity**						0.011					0.006
	Insufficient	14,008	(70.0)	5,996	(30.0)		13,461	(58.3)	9,621	(41.7)	
	Sufficient	4,553	(71.7)	1,797	(28.3)		1,289	(55.4)	1,039	(44.6)	
**Socioeconomic status**						< 0.001					< 0.001
	High	8,559	(72.6)	3,237	(27.4)		6,137	(61.1)	3,912	(38.9)	
	Middle	8,309	(71.0)	3,387	(29.0)		7,301	(58.7)	5,127	(41.3)	
	Low	1,693	(59.2)	1,169	(40.8)		1,312	(44.7)	1,621	(55.3)	
**Perceived health status**						< 0.001					< 0.001
	High	13,830	(76.7)	4,205	(23.3)		10,003	(55.5)	4,793	(26.6)	
	Middle	3,637	(62.1)	2,221	(37.9)		3,838	(65.5)	3,848	(65.7)	
	Low	1,094	(44.5)	1,367	(55.5)		909	(36.9)	2,019	(82.0)	
**Suicidality**						< 0.001					< 0.001
	Never	17,577	(75.4)	5,731	(24.6)		13,631	(65.9)	7,041	(34.1)	
	Ever	984	(32.3)	2,062	(67.7)		1,119	(23.6)	3,619	(76.4)	
**BMI** ^**a**^						0.002					0.006
	Underweight	1,318	(68.4)	610	(31.6)		1,386	(71.9)	1,042	(54.0)	
	Normal	12,068	(71.2)	4,878	(28.8)		10,606	(62.6)	7,468	(44.1)	
	Overweight	1,954	(70.3)	825	(29.7)		1,118	(40.2)	848	(30.5)	
	Obese	2,814	(68.7)	1,282	(31.3)		1,235	(30.2)	1,001	(24.4)	
**Total**		18,561	(70.4)	7,793	(29.6)		14,750	(58.0)	10,660	(42.0)	

*Abbreviation* GAD-7, Generalized anxiety disorder-7; BMI, body mass index

ªBMI: underweight (BMI < 5th percentile), normal (5th < BMI ≤ 85th percentiles), overweight (85th < BMI ≤ 95thpercentiles), and obese (BMI > 95th percentile)

Table 2 presents a multiple logistic regression analysis of the association between watching *mukbang* and GAD. Even after adjusting for various covariates, both male and female participants who watched *mukbang* showed a statistically significant higher likelihood of GAD occurrence (aOR: 1.100, 95% CI: 1.026–1.180, *P* = 0.008 in male participants, aOR: 1.090, 95% CI: 1.003–1.185, *P* = 0.042 in female participants).

**Table 2:** Results of factors associated between watching *mukbang* and GAD-7

Variables		Male (*N* = 26,350)			Female (*N* = 25,410)		
		GAD-7 ≥ 5			GAD-7 ≥ 5		
		aOR	95% CI	*P*-value	aOR	95% CI	*P*-value
**Mukbang and Cookbang**							
	Never watching	1.000			1.000		
	Ever watching	1.100	(1.026-1.180)	0.008	1.090	(1.003-1.185)	0.042
**Age**							
	12–15	1.000			1.000		
	16–18	1.075	(0.997-1.159)	0.059	0.850	(0.787-0.919)	< 0.0001
**Family structure**							
	Both parents	1.000			1.000		
	Single parent	0.971	(0.817-1.153)	0.734	0.908	(0.777-1.061)	0.223
	No parent	0.893	(0.820-0.972)	0.009	0.853	(0.779-0.935)	0.001
**Smoking status**							
	Non-smoker	1.000			1.000		
	Current smoker	1.005	(0.905-1.116)	0.921	1.225	(1.038-1.446)	0.017
**Alcohol status**							
	Non-drinker	1.000			1.000		
	Past drinker	1.024	(0.946-1.109)	0.556	1.191	(1.100-1.290)	< 0.0001
	Current drinker	1.217	(0.914-1.619)	0.178	1.424	(0.976-2.076)	0.066
**Academic achievement**							
	Low	1.000			1.000		
	Middle	0.951	(0.868-1.041)	0.275	1.032	(0.946-1.125)	0.480
	High	1.070	(0.977-1.173)	0.145	1.022	(0.936-1.117)	0.626
**Sleep duration**							
	More than 8 h	1.000			1.000		
	6–8 h	1.226	(1.069-1.406)	0.004	1.200	(1.02-1.412)	0.0002
	Less than 6 h	1.428	(1.251-1.629)	< 0.0001	1.360	(1.160-1.594)	0.028
**Perceived stress level**							
	Low	1.000			1.000		
	Middle	3.214	(2.826-3.655)	< 0.0001	3.618	(3.126-4.186)	< 0.0001
	High	11.735	(10.315-13.351)	< 0.0001	15.308	(13.333-17.575)	< 0.0001
**Physical activity**							
	Insufficient	1.000			1.000		
	Sufficient	1.015	(0.933-1.104)	0.731	1.067	(0.955-1.193)	0.251
**Socioeconomic status**							
	High	1.000			1.000		
	Middle	0.991	(0.920-1.068)	0.812	1.043	(0.968-0.968)	0.2662
	Low	1.221	(1.085-1.373)	0.001	1.305	(1.162-1.467)	< 0.0001
**Perceived health status**							
	High	1.000			1.000		
	Middle	1.580	(1.465-1.703)	< 0.0001	1.497	(1.393-1.609)	< 0.0001
	Low	2.476	(2.206-2.780)	< 0.0001	2.362	(2.095-2.664)	< 0.0001
**Suicidality**							
	Never	1.000			1.000		
	Ever	3.823	(3.462-4.222)	< 0.0001	3.293	(3.004-3.609)	< 0.0001
**BMI**							
	Underweight	0.998	(0.869-1.146)	0.978	0.982	(0.873-1.105)	0.765
	Normal	1.000			1.000		
	Overweight	0.909	(0.809-1.021)	0.107	0.922	(0.811-1.047)	0.211
	Obese	0.860	(0.783-0.946)	0.002	0.915	(0.816-1.026)	0.129

*Abbreviation* aOR, adjusted odds ratio; CI, confidence interval; GAD-7, Generalized anxiety disorder-7; BMI, body mass index

All variables except ‘Mukbang and Cookbang’ and ‘GAD-7’ are covariates

Table 3 shows the results of the subgroup analysis of the association between watching *mukbang* and GAD-7 stratified by independent variables. After dividing the adolescents based on whether they watched *mukbang*, most subgroups exhibited similar trends to the main results. Subgroups with high levels of perceived stress (aOR: 1.136, 95% CI: 1.027–1.257, P = 0.013 in male participants, aOR: 1.166, 95% CI: 1.040–1.307, P = 0.009 in female participants) and those that engaged in insufficient physical activity showed significantly higher odds ratio of GAD in the “Ever watching” group compared to the “Never watching” group.

**Table 3:** Subgroup analysis of the association between watching *mukbang* and GAD-7 stratified by independent variables

Variables		Never watching	Ever watching			Never watching	Ever watching		
			GAD-7 ≥ 5				GAD-7 ≥ 5		
		aOR	aOR	95% CI	*P*-value	aOR	aOR	95% CI	*P*-value
		**Boys (** ***N*** *** = 26,350)***				**Girls (** ***N*** *** = 25,410)***			
**Age**									
	12–15	1.000	1.131	(1.026-1.247)	0.014	1.000	1.080	(0.969-1.204)	0.166
	16–18	1.000	1.068	(0.965-1.182)	0.203	1.000	1.107	(0.618-1.007)	0.116
**Family structure**									
	Both parents	1.000	1.048	(0.959-1.144)	0.300	1.000	1.079	(0.982-1.185)	0.113
	Single parent	1.000	1.030	(0.726-1.461)	0.869	1.000	1.295	(0.854-1.965)	0.224
	No parent	1.000	1.240	(1.077-1.426)	0.003	1.000	1.101	(0.903-1.341)	0.340
**Alcohol status**									
	Non-drinker	1.000	1.148	(1.056-1.249)	0.001	1.000	1.112	(1.009-1.225)	0.032
	Past drinker	1.000	1.025	(0.908-1.157)	0.690	1.000	1.029	(0.869-1.219)	0.738
	Current drinker	1.000	1.334	(0.784-2.271)	0.287	1.000	1.291	(0.533-3.127)	0.572
**Smoking status**									
	Non-smoker	1.000	1.113	(1.033-1.200)	0.005	1.000	1.104	(1.014-1.202)	0.023
	Current smoker	1.000	1.000	(0.819-1.220)	0.998	1.000	0.831	(0.540-1.279)	0.400
**Academic achievement**									
	High	1.000	1.117	(0.996-1.251)	0.058	1.000	1.047	(0.923-1.189)	0.474
	Middle	1.000	1.109	(0.974-1.263)	0.119	1.000	1.194	(1.029-1.387)	0.020
	Low	1.000	1.074	(0.948-1.218)	0.260	1.000	1.053	(0.910-1.219)	0.485
**Sleep duration**									
	More than 8 h	1.000	0.941	(0.750-1.181)	0.598	1.000	1.144	(0.839-1.559)	0.395
	6–8 h	1.000	1.290	(1.145-1.454)	< 0.0001	1.000	1.054	(0.905-1.228)	0.495
	Less than 6 h	1.000	1.023	(0.929-1.127)	0.638	1.000	1.101	(0.992-1.223)	0.071
**Perceived stress level**									
	High	1.000	1.136	(1.027-1.257)	0.013	1.000	1.166	(1.040-1.307)	0.009
	Middle	1.000	1.048	(0.936-1.175)	0.415	1.000	0.992	(0.865-1.138)	0.912
	Low	1.000	1.135	(0.886-1.453)	0.315	1.000	1.127	(0.789-1.611)	0.511
**Physical activity**									
	Insufficient	1.000	1.095	(1.010-1.187)	0.028	1.000	1.101	(1.008-1.202)	0.032
	Sufficient	1.000	1.124	(0.958-1.318)	0.150	1.000	0.999	(0.759-1.315)	0.996
**Socioeconomic status**									
	High	1.000	1.100	(0.992-1.220)	0.070	1.000	1.182	(1.034-1.352)	0.015
	Middle	1.000	1.136	(1.016-1.269)	0.025	1.000	0.985	(0.872-1.112)	0.806
	Low	1.000	0.957	(0.780-1.174)	0.670	1.000	1.206	(0.954-1.524)	0.117
**Perceived health status**									
	High	1.000	1.101	(1.000-1.211)	0.051	1.000	1.164	(1.038-1.304)	0.010
	Middle	1.000	1.117	(0.977-1.276)	0.105	1.000	1.043	(0.901-1.209)	0.571
	Low	1.000	1.048	(0.849-1.293)	0.663	1.000	0.908	(0.712-1.158)	0.437
**Suicidality**									
	Never	1.000	1.123	(1.039-1.212)	0.003	1.000	1.049	(0.956-1.150)	0.314
	Ever	1.000	0.948	(0.792-1.135)	0.562	1.000	1.293	(1.041-1.605)	0.669
**BMI**									
	Underweight	1.000	1.090	(0.845-1.405)	0.507	1.000	1.021	(0.796-1.309)	0.872
	Normal	1.000	1.089	(1.000-1.184)	0.049	1.000	1.106	(1.006-1.217)	0.038
	Overweight	1.000	1.173	(0.935-1.472)	0.167	1.000	1.036	(0.772-1.390)	0.814
	Obese	1.000	1.107	(0.926-1.324)	0.264	1.000	1.110	(0.840-1.467)	0.463

*Abbreviation* aOR, adjusted odds ratio; CI, confidence interval, GAD-7, Generalized anxiety disorder-7; BMI, body mass index

All variables except ‘Mukbang and Cookbang’ and ‘GAD-7’ are covariates

**Fig. 2:**
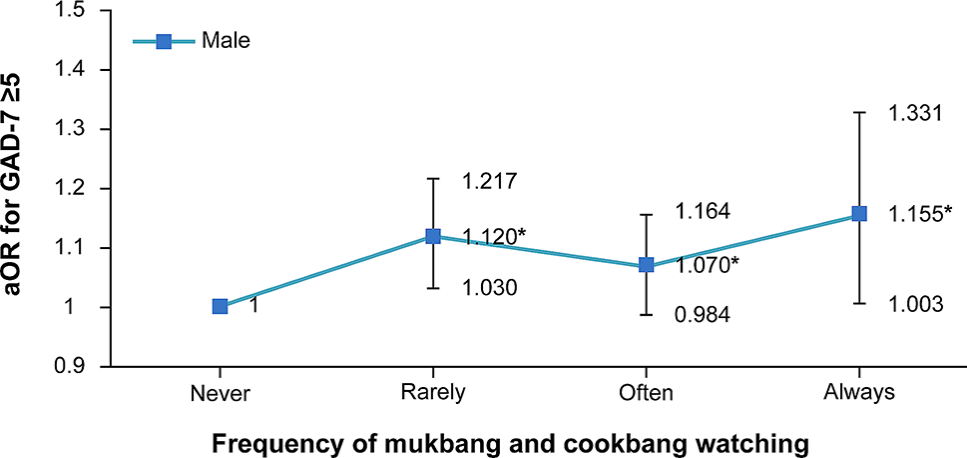
Association between *mukbang* and *cookbang* watching categorized by frequency and GAD-7 among male participants, where “Rarely” is less than once a week, “Often” is less than seven times a week), “Always” is every day

Figure 2 shows the results of subgroup analysis for male participants based on their frequency of watching *mukbang*. The analysis showed that among those who always watched *mukbang*, the likelihood of GAD increased compared to participants who never watched *mukbang* (aOR: 1.155, 95% CI: 1.003–1.331, p-for-trend = 0.0291).

**Fig. 3:**
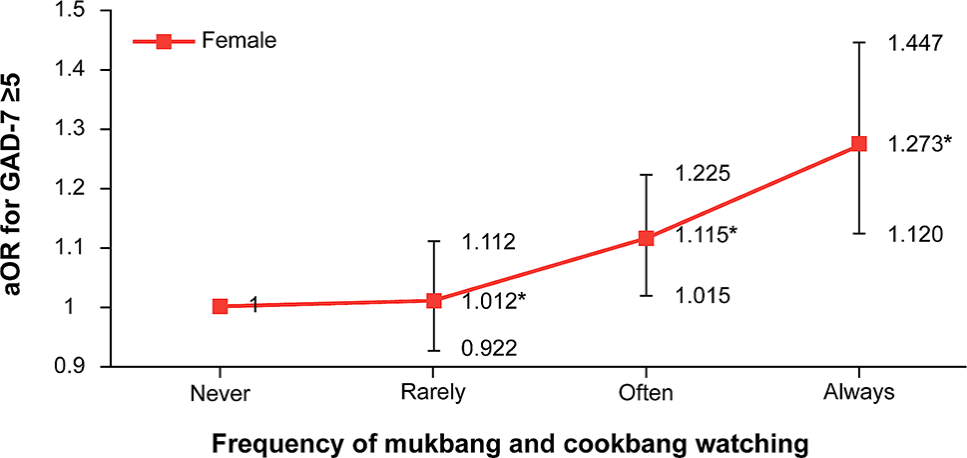
Association between *mukbang* and *cookbang* watching categorized by frequency and GAD-7 among female participants, where “Rarely” is less than once a week, “Often” is less than seven times a week, and “Always” is every day

Figure 3 shows the results of subgroup analysis for female participants based on their frequency of watching *mukbang*. The analysis showed that among those who always watched *mukbang*, the likelihood of GAD increased compared with participants who never watched *mukbang* (aOR: 1.273, CI: 1.120–1.447, p-for-trend < 0.0001). As the frequency of watching *mukbang* increased, the likelihood of GAD among female participants increased compared to those who never watched *mukbang*. A dose-dependent relationship was observed between the frequency of watching *mukbang* and the likelihood of GAD among female adolescents.

## Discussion

This study confirmed that watching *mukbang* is associated with GAD among Korean adolescents. Even after adjusting for various covariates and stratifying by sociodemographic, health-related, and psychosocial factors, the association persisted. Moreover, we found a dose-dependent relationship between the frequency of *mukbang* watching and GAD in female adolescents.

Eating disorders are an important public health issue characterized by disturbances in eating patterns and thoughts related to eating, food, body weight and shape [26]. The type of eating disorders include bulimia nervosa, binge-eating disorder, and anorexia nervosa [27]. Previous research has shown a correlation between watching mukbang and disordered eating behaviors [28]. Famous mukbang creators typically prepare and consume meals with excessive calories, but it is common in mukbang video to see slender hosts eating large quantities of food [9]. Such discrepancies, like slim hosts consuming large amounts of food, can lead to distorted body image, body dissatisfaction, and disordered eating behaviors among viewers [29].

Our results tend to align with research that shows problematic online behaviors, including excessive *mukbang* watching, are associated with difficulties in regulating emotions like anxiety [30–33]. Adolescence is the transitional and developmental period from childhood to adulthood [34]. Detecting and evaluating anxiety disorders in adolescents is challenging but necessary [35]. Protecting adolescents from anxiety disorders is important because they can lead to adult anxiety and potentially lifelong mental health issues [36, 37]. Male individuals were more vulnerable to media and Internet addiction [38]. The relationship between watching *mukbang* and anxiety disorders has shown differences based on gender. In the result, the likelihood of having GAD was higher in male adolescents (Table 2). Both male and female adolescents who watched *mukbang* and also had other bad habits, including insufficient physical activity or high perceived stress levels, were more likely to have GAD (Table 3). Female individuals experience more stress regarding eating than male individuals do after consuming food-related content [39]. In this study, these differences were more pronounced in female than in male participants. After adjustment, female participants tended to have higher perceived stress levels than male participants (Table 3). When anxiety disorders are associated with bad habits, watching excessive *mukbang* could potentially exacerbate anxiety disorders [40]. Therefore, regulating adolescents’ viewing of *mukbang* and considering the limitations of viewing time and content are warranted.

The mechanism underlying the relationship between *mukbang* watching and GAD remains unclear. Several hypotheses have been proposed. First, impulsive behaviors associated with watching *mukbang* may lead to GAD [41]. These behaviors include eating quickly or in large quantities, imitating foods seen in programs, eating snacks instead of regular meals, and consuming stimulating foods [42]. Second, watching *mukbang* may influence GAD in individuals with difficulties in emotion regulation. Individuals with poor emotion regulation seem to use online activities to control and regulate their emotional experiences, and *mukbang* watching may be one of these activities [43]. Third, there is a possibility that *mukbang* could aggravate psychological distress. *Mukbang* watching provides social comfort by showing individuals in videos producing satisfactory sounds and visuals while enjoying food and interacting with viewers. However, excessive *mukbang* watching can potentially worsen the psychological distress in individuals with anxiety disorder, as it may develop into problematic online behavior that attempts to avoid anxiety about real-life issues [44]. Furthermore, watching *mukbang* may contribute to anxiety by reducing participation in other activities, such as physical activities or social interactions [45]. Those who watch *mukbang* frequently and for longer periods at a time are more likely to exhibit tendencies toward obesity. Obesity is both a risk factor for anxiety and the result of a dietary calorie imbalance [46, 47]. Adolescents with anxiety may, therefore, be more inclined to watching *mukbang* [48].

Previous studies have identified several beneficial uses of recreational *mukbang* watching, *Mukbang* has become popular among individuals dissatisfied with their real lives, who use the internet and social media as a way of experiencing immediate satisfaction and escaping from reality [8]. *Mukbang* provides entertainment and relaxation by virtually consuming harmful foods without experiencing actual health consequences [10]. Additionally, watching *mukbang* allows people who eat or prepare meals alone to reduce their feelings of loneliness by engaging in conversations with online *mukbang* hosts [49, 50].

This study had some limitations. First, cross-sectional data is a cross-sectional study, so it is difficult to establish a causal relationship between watching *mukbang* and GAD, or to determine the impact of watching *mukbang* on GAD. Additional studies using experimental or longitudinal designs are needed to establish causality. Second, self-reports may diverge from legitimate information due to recall bias and the potential for participant misunderstanding. Third, there is a lack of objective scales for measuring certain variables such as specific details of *mukbang*, including daily content and viewing data on *mukbang*.

Despite these limitations, our study has certain strengths. The KYRBS includes nationally representative data, allowing the study results to be generalized to the entire population of Korean adolescents. These findings could be beneficial for public health policies related to the impact of media on adolescent mental health. Ultimately, further research is needed to define the causal relationship between watching *Mukbang* and GAD, and to investigate the underlying mechanisms.

## Conclusion

This study found that adolescents who watched *mukbang* may be more susceptible to GAD, with a dose-dependent relationship observed in female individuals. Therefore, proper interventions for mental health are needed for adolescents who watch *mukbang*.

### Electronic supplementary material

Below is the link to the electronic supplementary material.

Supplementary Material 1

## Data Availability

The data analyzed in this study were taken from the 2022 KYRBS which is available to the public. All data can be downloaded from the KYRBS official website (https://www.kdca.go.kr/yhs/).
