# Supplementary material for: Association between watching eating broadcasts like mukbang and cookbang and generalized anxiety disorder among Korean adolescents
Source: BMC Psychiatry. 2024 Jul 30;24:536. doi: 10.1186/s12888-024-05957-z (PMC11290270; doi:10.1186/s12888-024-05957-z)
Supplement: Supplementary file 1 — Supplementary Material 1 [file 12888_2024_5957_MOESM1_ESM.docx]

**Additional file 1. Mukbang and cookbang questions**

34. How frequently did you watch mukbang (eating broadcasts) and cookbang (cooking broadcasts) in the past 12 months?

※"Mukbang" is a shortened term for "eating broadcast," and "cookbang" is a shortened term for "cooking broadcast." These refer to content on social media (Instagram, Facebook, TikTok, etc.), TV programs, and internet broadcasts (YouTube, AfreecaTV, KakaoTV, Twitch, etc.).

①None

② Less than once a month

③ More than once a month and less than three times a month

④ Once or twice a week

⑤ Three or four times a week

⑥ Five or six times a week

⑦ Every day

**Additional file 2. GAD-7(Generalized Anxiety Disorder-7) questions**

45. How often have you been disturbed by the following issues in the past two weeks?

| GAD-7 Questions | ① Not at all | ② Several days | ③ More than half the days | ④ Nearly every day |  |
| --- | --- | --- | --- | --- | --- |
| 1) Feeling nervous, anxious or on edge |  |  |  |  |  |
| 2) Not being able to stop or control worrying |  |  |  |  |  |
| 3) Worrying too much about different things |  |  |  |  |  |
| 4) Trouble relaxing |  |  |  |  |  |
| 5) Being so restless that it is hard to sit still |  |  |  |  |  |
| 6) Becoming easily annoyed or irritable |  |  |  |  |  |
| 7) Feeling afraid as if something awful |  |  |  |  |  |
